# Proposed risk factors for infection with multidrug-resistant pathogens in hemodialysis patients hospitalized with pneumonia

**DOI:** 10.1186/s12879-017-2788-8

**Published:** 2017-10-12

**Authors:** Jae-Uk Song, Hye Kyeong Park, Hyung Koo Kang, Jonghoo Lee

**Affiliations:** 1 Division of Pulmonary and Critical Care Medicine, Department of Internal Medicine, Kangbuk Samsung Hospital, Sungkyunkwan University School of Medicine, Seoul, South Korea; 2 Division of Pulmonary and Critical Care Medicine, Department of Internal Medicine, Ilsan Paik Hospital, Inje University College of Medicine, Goyang-si, South Korea; 3 Department of Internal Medicine, Jeju National University Hospital, Jeju National University School of Medicine, Aran 13 gil 15, Jeju-si, Jeju Special Self-Governing Province 690-767 South Korea

**Keywords:** Pneumonia, Hemodialysis, End-stage renal disease, Multidrug resistance, Pathogen

## Abstract

**Background:**

In patients with hemodialysis-associated pneumonia (HDAP), information on both microbiologic features and antimicrobial strategies is limited. The aim of this study is to investigate predictive factors of infection with multidrug-resistant (MDR) pathogens in HDAP patients.

**Methods:**

This was a multicenter, retrospective, and observational study. Enrolled patients were classified into MDR or non-MDR pathogens groups according to culture results. We examined risk factors of infection with MDR pathogens and created a decision support tool using these risk factors.

**Results:**

MDR pathogens were identified in 24 (22.8%) out of a total of 105 HDAP patients. The most common MDR pathogens were methicillin-resistant *Staphylococcus aureus* (10 patients, 9.5%) and the isolation rate of *Pseudomonas aeruginosa* was 6.6%. Logistic regression showed two variables were associated with the isolation of MDR pathogens: recent hospitalization (adjusted odds ratio [OR]: 2.951, 95% confidence interval [CI]: 1.022–8.518) and PSI (Pneumonia Severity Index) score (adjusted OR: 1.023, 95% CI: 1.005–1.041). The optimal cut-off value for PSI score using a receiver operating characteristic curve analysis was 147. According to the presence of 0, 1, or 2 of the identified risk factors, the prevalence of MDR pathogens was 7.6, 28.2 and 64.2%, respectively (*p* < 0.001 for trend). The area under the curve of the prediction tool was 0.764 (95% CI: 0.652–0.875).

**Conclusions:**

We demonstrated that recent hospitalization and PSI > 147 are risk factors of infection with MDR pathogens in HDAP patients. This simple proposed tool would facilitate more accurate identification of MDR pathogens in these patients.

## Background

In hemodialysis (HD) populations, pneumonia is common and a leading cause of death [1, 2]. Because of the uremic internal milieu and the very frequent coexistence of serious comorbid medical conditions, these patients can be considered chronically immunosuppressed [3]. According to the United States Renal Dada System (USRDS) registry, approximately 20% of patients developed pneumonia in the 1-year period following initiation of dialysis therapy [1]. The mortality rates from pneumonia in hemodialysis (HD) patients were higher than those from pneumonia in the general population [2]. Therefore, early proper management is important to reduce mortality in HD patients with pneumonia.

Until now, there are no guidelines focused primarily on hemodialysis-associated pneumonia (HDAP). Because the 2005 American Thoracic Society (ATS)/Infectious Diseases Society of America (IDSA) guidelines included HDAP as a category of HCAP, HDAP patients could receive broad-spectrum antibiotics targeted against multidrug-resistant (MDR) pathogens [4]. But, several studies demonstrated that HCAP does not always identify MDR pathogens [5]. The 2016 ATS/IDSA guidelines removed the concept of HCAP among a category of nosocomial pneumonia [6].

The clinical epidemiology of HDAP has received little attention to date [7]. A previous study including some data used in the present study revealed that the HDAP group was clinically more similar to the CAP group than to the HCAP other than HDAP (O-HCAP) [7]. Accordingly, whether MDR pathogens-targeted antibiotics should be selected in patients with HDAP is unclear. Because of the uncertainty surrounding the actual risks of infection with MDR pathogens in HDAP patients and the increasing burden of end-stage renal disease worldwide [8], more data are required for a better distinct targeted therapeutic approach. Therefore, we investigated microbiologic characteristics and novel predictive factors of infection with MDR pathogens in patients hospitalized with HDAP. We also developed a prediction tool using these risk factors to identify subjects infected with MDR pathogens.

## Methods

### Study design, populations, and recorded parameters

We retrospectively conducted observational cohort studies at three institutions (Jeju National University Hospital, Kangbuk Samsung Hospital, and Ilsan Paik Hospital) between January 2011 and December 2015. Some of the clinical data for patients enrolled at Jeju National University Hospital were included in an article published in 2016 [7].

Patients were screened by the Korean Standard Classification of Diseases-7 codes of the followings; J18.0–18.9 as representative codes of pneumonia in the primary discharge diagnosis and N18.5, N18.9, or Z49.1 as codes of HD [9]. The medical records and radiological findings were reviewed to confirm the diagnosis of pneumonia by the following criteria: the presence of a new infiltrate on chest radiography with symptoms and signs of a lower respiratory tract infection. And patients on regular intermittent HD 3 times a week were included in the analysis. We excluded the following types of patients: (1) those who did not receive dialysis at the time of admission, (2) those who underwent continuous renal replacement therapy after organ failure developed by pneumonia, (3) those who had hospital-acquired pneumonia (HAP) developed at least 48 h after hospital admission, (4) those who did receive continuous ambulatory peritoneal dialysis, (5) those who re-visited within 10 days of discharging, and (6) those who transferred from other hospitals after hospitalization for >48 h.

According to culture results, enrolled patients were classified as MDR or non-MDR pathogens groups. We compared clinical characteristics, severity of pneumonia, identified pathogens, antibiotics, and clinical outcomes between the two groups. The severity of pneumonia was assessed by the CURB-65 (confusion, urea, respiratory rate, blood pressure, age more than 65 years) and Pneumonia Severity Index (PSI) scores [10, 11].

### Definitions

HDAP was defined as pneumonia developing in patients receiving chronic HD within 30 days. O-HCAP was defined using the criteria of the 2005 ATS/IDSA guidelines as follows: recent history of hospitalization in an acute care hospital for ≥2 days in the past 90 days; residence in a nursing home or long-term care facility (NHAP, nursing home-acquired pneumonia); or recent outpatient intravenous therapy or wound care within the past 30 days [4]. Severe pneumonia was defined according to ATS/IDSA 2007 criteria [12].

In accordance with the 2005 ATS/IDSA guidelines [4], methicillin-resistant *Staphylococcus aureus* (MRSA), *Pseudomonas aeruginosa*, extended-spectrum beta-lactamase (ESBL)-producing or carbapenem-resistant *Klebsiella pneumoniae* and *Escherichia coli, Acinetobacter baumanii*, and *Stenotrophomonas maltophilia* were considered to be MDR pathogens. According to susceptibility test criteria for lower respiratory tract pathogens, the appropriateness of antibiotic therapy was analyzed for all cases with an etiological diagnosis. Inappropriate antibiotic therapy was defined if the empirical antibiotics were not effective or unnecessarily broad against the identified pathogens based on in vitro susceptibility testing [13]. Failure of initial antibiotics therapy was defined as death during initial treatment or change of antibiotics from initial agents to others after 48 h due to clinical instability [14].

### Microbiology

Sputum, tracheal aspirate, bronchial alveolar lavage fluid or blood samples were investigated for microbial analysis. Respiratory samples were cultured in a semi-quantitative manner, and pathogens were identified when a predominant microorganism was detected from group 4 or 5 sputum, according to Geckler’s grading system [15]. Blood cultures were considered as pathogens if there was no other infection source for a positive blood culture. Paired serology for *Mycoplasma pneumoniae* or *Chlamydia pneumoniae* and urinary antigen tests for *Streptococcus pneumoniae* and *Legionella pneumophila* serogroup 1 were also recorded if these exams were checked. The antibiotic sensitivity of all isolates was determined using a disc diffusion method, according to the Clinical and Laboratory Standards Institute guidelines [16].

### Statistical analyses

Data are presented as medians and interquartile ranges (IQRs; 25th and 75th percentiles) for continuous variables and as numbers and percentages for categorical variables. For comparison of continuous variables, the Mann-Whitney U-test between two groups and the Kruskal-Wallis test among three groups were used to compare the median values. Categorical variables were compared using the Pearson χ^2^ test, and the Fisher’s exact test was used when any cell contained fewer than five data points.

To identify independent predictive factors associated with occurrence of MDR pathogens, we performed multivariate logistic regression analyses, as measured by the estimated odds ratio (OR) with 95% confidence interval (CI). Potential candidate variables with a *P*-value less than 0.05 in univariate analysis were entered into the regression model. From logistic regression results, we created predictive tool to identify patients with HDAP due to MDR pathogens. We classified patients based on the presence of risk factors for MDR pathogens. Then, we evaluated the predictive value of the proposed support tool for correctly indicating the presence of infection with MDR pathogens via a receiver operating characteristic (ROC) curve. The estimated area under the ROC curve (AUC) values were compared using the Hanley-McNeil test [17]. The cut-off point that showed the highest Youden Index was considered the optimal cut-off value [18]. All tests were two-sided, and *P*-values <0.05 were considered statistically significant. All analyses were performed using the Statistical Package for the Social Sciences (SPSS) network version 18.0 (SPSS; Chicago, IL, USA).

## Results

### Baseline characteristics and clinical outcomes

**Fig. 1:**
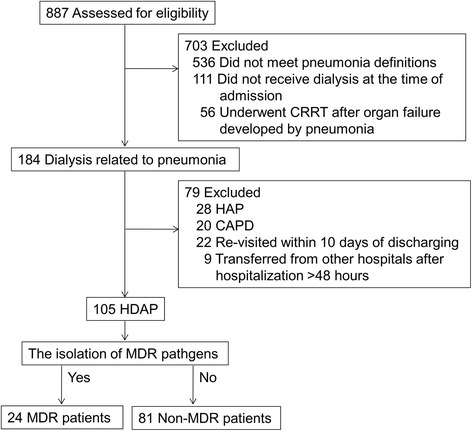
Patient flow. CRRT, continuous renal replacement therapy; HAP, hospital-acquired pneumonia; CAPD, continuous ambulatory peritoneal dialysis; HDAP, hemodialysis-associated pneumonia; MDR, multidrug-resistant

A total of 887 patients were initially identified through medical records. Of these patients, 703 were excluded for the reasons in the followings; 536 did not meet pneumonia definitions, 111 did not receive dialysis at the time of admission, and 56 underwent CCRT after organ failure developed by pneumonia (Fig. 1). A total of 184 patients (21.1%) were enrolled as the dialysis related to pneumonia. Seventy-nine patients were excluded for the reasons presented in Fig. 1. Finally, a total of 105 patients were included in the present study.

There were 68 males and 37 females, with a median age of 71 years. MDR pathogens were identified in 24 patients (22.8%). The baseline clinical characteristics and clinical outcomes of the total 105 HDAP patients are summarized in Table 1.

**Table 1:** Baseline clinical characteristics and treatment outcomes of patients admitted with hemodialysis-associated pneumonia

Characteristics	Overall patients (*n* = 105)	MDR pathogens group (*n* = 24)	Non-MDR pathogens group (*n* = 81)	*P* Value
Age (years)	71 (61–76)	73 (62–79)	71 (61–76)	0.199
Male	68 (64.8%)	15 (62.5%)	53 (65.4%)	0.792
Female	37 (35.2%)	9 (37.5)	28 (34.6%)	0.792
Time interval between dialysis and pneumonia (months)	30 (11–69)	21 (10–74)	34 (12–69)	0.541
Etiology of dialysis^a^				
Diabetes mellitus	60 (57.1%)	14 (58.3%)	46 (56.7%)	0.344
Hypertension	57 (54.2%)	11 (45.8%)	46 (56.7%)	0.893
Glomerulonephropathy	6 (5.7%)	1 (4.1%)	5 (6.1%)	1.000
Idiopathic	7 (6.6%)	2 (8.3%)	5 (6.1%)	0.658
Others	13 (12.3%)	1 (4.1%)	12 (14.8%)	0.289
Tube feeding	9 (8.5%)	5 (20.8%)	4 (4.9%)	0.028
HCAP criteria other than HDAP		17 (70.8%)	27 (33.3%)	0.001
Recent hospitalization	42 (40.0%)	16 (66.6%)	26 (32.0%)	0.002
NHAP	10 (9.5%)	5 (20.8%)	5 (6.1%)	0.047
Recent intravenous therapy	10 (9.5%)	3 (12.5%)	7 (8.6%)	0.692
Clinical parameters				
Severe pneumonia	37 (35.2%)	13 (54.1%)	24 (29.6%)	0.027
Confusion	15 (14.2%)	6 (25.0%)	9 (11.1%)	0.103
Respiratory failure	47 (44.7%)	14 (58.3%)	33 (40.7%)	0.128
Sepsis or septic shock at onset	15 (14.2%)	7 (29.1%)	8 (9.8%)	0.040
ICU admission	22 (20.9%)	10 (41.6%)	12 (14.8%)	0.005
Need for ventilator	6 (5.7%)	3 (12.5%)	3 (3.7%)	0.131
Radiological findings				
Multi-lobar involvement	73 (69.5%)	20 (83.3%)	53 (65.4%)	0.094
Pleural effusion	33 (31.4%)	6 (25.0%)	27 (33.3%)	0.440
Laboratory findings				
WBC (/mm^3^)	11,200 (7400–15,015)	13,210 (8200–18,600)	10,680 (7150–14,960)	0.104
CRP (mg/dl)	8.5 (3.7–15.1)	9.4 (4.8–16.0)	7.1 (3.7–14.7)	0.364
Procalcitonin, *n* = 62, (mg/dl)	1.1 (0.4–5.9)	1.7 (0.5–6.8)	0.9 (0.3–5.6)	0.571
Indices for disease severity				
CURB-65 score	2 (1–2)	2 (1–3)	2 (1–2)	0.095
CURB-65 score ≥ 3	19 (18.0%)	7 (29.1%)	12 (14.8%)	0.133
PSI score	123 (105–145)	148 (120–181)	118 (99–139)	0.001
PSI class IV or V	91 (86.6%)	23 (95.8%)	68 (83.9%)	0.181
Initial antibiotic therapy				
as CAP	47 (44.7%)	4 (16.6%)	43 (53.0%)	0.002
as HAP	58 (55.2%)	20 (83.3%)	38 (46.9%)	0.002
Use of Anti-MRSA agents	7 (6.6%)	4 (16.6%)	3 (3.7%)	0.046
Clinical outcomes				
Use of inappropriate antibiotics	21 (20.0%)	15 (62.5%)	6 (7.4%)	<0.001
Change of initial antibiotics	40 (38.0%)	13 (54.1%)	27 (33.3%)	0.065
Failure of initial antibiotics therapy	29 (27.6%)	11 (45.8%)	18 (22.2%)	0.254
Duration of antibiotic therapy (days)	12 (10–15)	12 (9–22)	12 (10–15)	0.401
Length of hospital stay (days)	11 (7–17)	14 (9–25)	11 (7–16)	0.093
Pneumonia-related mortality rate	8 (7.6%)	6 (25.0%)	2 (2.4%)	0.002
Hospital mortality rate	11 (10.4%)	6 (25.0%)	5 (6.1%)	0.016

Data are presented as median (interquartile range) or number (%)

*MDR* multidrug-resistant, *HCAP* healthcare-associated pneumonia, *HDAP* hemodialysis-associated pneumonia, *NHAP* nursing home-acquired pneumonia, *ICU* intensive care unit, *WBC* white blood cell, *CRP* C-reactive protein, *CAP* community-acquired pneumonia, *MRSA* methicillin-resistant *Staphylococcus aureus*, *CURB-65* Confusion, Urea, Respiratory rate, Blood pressure, Age ≥ 65, *PSI* Pneumonia Severity Index

^a^allowed for overlap

### Microbiological etiology

Table 2 shows the distribution of causative organisms. Of the total 105 HDAP patients, the responsible pathogens were determined only in 53 patients (50.4%). The most common pathogen was *S. aureus* (17, 16.1%), which consisted of methicillin-sensitive *S. aureus* (7, 6.6%) and MRSA (10, 9.5%), followed by *K. pneumoniae* (11, 10.4%) and *S. pneumoniae* (10, 9.5%). The isolation rates of drug-resistant gram-negative bacteria *P. aeruginosa*, *A. baumanii*, ESBL-producing *K. pneumoniae* were 6.6, 5.7, and 2.8%, respectively.

**Table 2:** Microorganisms identified in patients admitted with hemodialysis-associated pneumonia

Microorganisms^a^	No. of patients (%)
Identified microorganisms^b^ Gram-positive bacteria	53 (50.4%)
*Streptococcus pneumoniae*	10 (9.5%)
*Staphylococcus aureus*	17 (16.1%)
MSSA	7 (6.6%)
MRSA	10 (9.5%)
Gram-negative bacteria	
*Pseudomonas aeruginosa*	7 (6.6%)
*Haemophilus influenza*	0 (0%)
*Klebsiella pneumoniae*	11 (10.4%)
ESBL (+)	3 (2.8%)
ESBL (−)	8 (7.6%)
*Acinetobacter baumannii*	6 (5.7%)
*Stenotrophomonas maltophilia*	1 (0.9%)
*Mycoplasma pneumonia*	2 (1.9%)
Other gram-negative species^c^	5 (4.7%)
Polymicrobial pathogens	8 (7.6%)
Multidrug-resistant pathogens^d^	24 (22.8%)

Data are presented as number (%)

HDAP: hemodialysis-associated pneumonia; MSSA: methicillin-sensitive *Staphylococcus aureus*; MRSA: methicillin-resistant *Staphylococcus aureus*

^a^Allowed for overlap

^b^One or more pathogens may be listed

^c^Other gram-negative species include *Escherichia coli, Enterobacter species, Serratia marcescens,* and *Legionella pneumophilia*

^d^Multidrug-resistant pathogens include Methicillin-resistant *Staphylococcus aureus* (MRSA), *Pseudomonas* species, *Acinetobacter* species, *Stenotrophomonas maltophilia*, and extended-spectrum β-lactamase (ESBL)-producing Enterobacteriaceae

### Predictive factors associated with occurrence of MDR pathogens

**Fig. 2:**
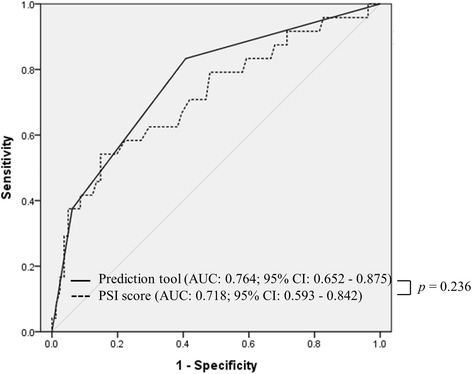
Comparison of receiver-operating characteristic (ROC) curves between the proposed tool and PSI score to predict infection with multidrug-resistant pathogens. PSI, pneumonia severity index

Table 3 shows multivariate logistic regression analyses of the four risk factors for MDR pathogens: tube feeding, recent hospitalization, NHAP, and PSI score. Recent hospitalization and PSI score was independently associated with the isolation of MDR pathogens in HDAP patients (adjusted OR: 2.951, 95% CI: 1.022–8.518, *p* = 0.045 and adjusted OR: 1.023, 95% CI: 1.005–1.041, *p* = 0.011, respectively). ROC curve analysis was used to assess optimal cutoff values for PSI score. The maximum sum of sensitivity and specificity was 147 for PSI (sensitivity 54.1%, specificity 85.1%, positive predictive value 52.0%, and negative predictive value 86.2%, Fig. 2).

**Table 3:** Multivariate logistic regression analysis for predictive factors associated with multidrug-resistant pathogens in patients admitted with hemodialysis-associated pneumonia

Risk factors	Odds ratio (95% confidence interval)	*P* Value
Tube feeding	2.229 (0.459–10.819)	0.320
Recent hospitalization	2.951 (1.022–8.518)	0.045
NHAP	3.535 (0.823–15.183)	0.090
PSI score	1.023 (1.005–1.041)	0.011

*NHAP* nursing home-acquired pneumonia, *PSI* Pneumonia Severity Index

### Proposed decision support tool for prediction of MDR pathogens

Based on the multivariate logistic regression analysis of the association with occurrence of MDR pathogens in patients with HDAP, PSI score > 147 and recent hospitalization were considered as predictive MDR risk factors. We created a decision support tool to predict MDR pathogens. Patients divided into low (without any risk factors)-, intermediate (with only one risk factor) -, and high (with both two risk factors)-risk strata based on two predictive MDR risk factors.

**Fig. 3:**
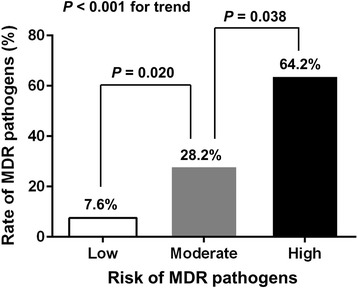
The probability of MDR pathogens stratified by risk using the prediction tool. MDR, multidrug-resistant

The ROC curves for prediction tool and PSI score are shown in Fig. 2 The prediction tool had a higher discriminatory power to identify MDR pathogen infection than PSI score, although there was no statistically significant difference (*p* = 0.228). The area under the curve (AUC) of the prediction tool (AUC: 0.764, 95% CI: 0.652–0.875, *p* < 0.001) tended to be greater than that of the PSI score (AUC: 0.718, 95% CI: 0.593–0.842, *p* = 0.001) (*p* = 0.236). The optimal cutoff for the prediction tool was 1 (sensitivity 83.3%, specificity 59.3%, positive predictive value 37.7%, and negative predictive value 92.3%). According to the risk stratification based on number of MDR risk variables, the prevalence of MDR pathogens was 7.6, 28.2 and 64.2%, respectively (p < 0.001 for trend, Table 4 and Fig. 3).

**Table 4:** Proposed prediction tools for multidrug-resistant pathogens in patients admitted with hemodialysis-associated pneumonia

Risk of MDR pathogens	Predictive factors	No. of patients	No. (%) of patients isolated with MDR pathogens
Low	PSI score ≤ 147 and no recent hospitalization	52	4 (7.6%)
Moderate	PSI score > 147 or recent hospitalization	39	11 (28.2%)
High	PSI score > 147 and recent hospitalization	14	9 (64.2%)

*MDR* multidrug-resistant, *PSI* Pneumonia: Severity Index

## Discussion

The current study revealed that 22.8% of hospitalized patients with HDAP had MDR pathogens. We also demonstrated that the occurrence of MDR pathogens was significantly associated with recent hospitalization within 3 months and PSI score more than 147. On the basis of these findings, we proposed a simple predictive tool to determine the risk of infection with MDR pathogens in HDAP using the number of risk factors. As the number of risk factors increased, the prevalence of infection with MDR pathogens also increased (low - 7.6%, intermediate - 28.2% and high - 64.2%, respectively; *p* < 0.001 for trend). Overall, this model had moderate predictive value, as demonstrated by the ROC curve (AUC: 0.764, 95% CI: 0.652–0.875). To our knowledge, this is the first study that proposes evidence based tool to predict infection with MDR pathogens among HDAP patients.

Previous studies have demonstrated that the isolation rate of MDR pathogens shows interregional differences in HCAP patients [5]. Also, HCAP consists of very heterogeneous subgroups [4], and there is little evidence that all criteria for HCAP convey similar risks for infection with MDR pathogens. This contributed to the removal of the concept of HCAP in the new 2016 ATS/IDSA guidelines for management of HAP and ventilator-associated pneumonia [6]. But, the concept of HCAP as a separate clinical entity would be still valid, because frequent interactions with the healthcare system can have potential risk for MDR pathogens [6]. Discordant results about the isolated rate of MDR pathogens among previous studies may be caused by the fact that the concept of HCAP includes various criteria for heterogeneous conditions [4], which did not have similar risks for infection with MDR pathogens [19]. Among the category of HCAP, only NHAP have been studied considerably, and MDR pathogens were not frequently isolated in these patients [20–25]. In contrast, the previous studies on HCAP included a relatively small proportion of 2.5~10.4% of HDAP patients [7, 13, 26–29]. Therefore, it is not known whether it is desirable to actively apply the guideline-concordant treatment to all patients with HDAP [4].

There are few studies focusing on the actual risk of MDR pneumonia in HDAP patients, resulting in a lack of microbiologic data on HDAP as a different category of HCAP. Although most HD patients live outside of hospitals, they manifest various degrees of immunodeficiency, regularly visit the hospital, and receive ongoing healthcare more often than non-HD patients. Compared to the general population, these characteristics of HD patients may contribute to the high incidence of MDR pathogens, which is not related to HD itself. Therefore, there is a question regarding whether all patients with HDAP should receive antibiotic therapy against MDR pathogens [30, 31].

We found two large cohort studies using the USRDS registry for clinical epidemiology of HDAP patients and four retrospective studies applying to the concept of HDAP [1, 2, 7, 30–32]. Unfortunately, in large cohort studies, the microorganisms in more than 80% of the patients hospitalized with HDAP could not be identified [1, 2]. The detection rate of *P. aeruginosa* was about 2%, but the rate of total isolated MDR pathogens was not mentioned in either study [1, 2]. In addition, four retrospective studies have demonstrated inconsistent MDR pathogen distributions in HDAP patients [7, 30–32]. In line with previous HDAP studies, the rate of isolated MDR pathogens was 5.6 to 35.4%, although microorganisms could not be identified in most patients. The detection rates of MRSA, *P. aeruginosa*, and *A. baumanii* were 0%~27.5%, 1.6%~16.7%, and 0%~4.2%, respectively [7, 30–32]. In the present study, MDR pathogens were identified in 22.8% of cases; of these, the most frequent microorganisms were MRSA (9.5%), followed by *P. aeruginosa* (6.6%), *A. baumanii* (5.7%). However, the rates of isolated MDR pathogens in previous studies and this study revealed a variable incidence range [7, 30–32].

Although we cannot offer satisfactory explanations for these various incidence rates of MDR pathogen distribution, these discordant results may be related to whether the selected populations included the other criteria for HCAP. The studies demonstrating relatively low MDR pathogen infections included patients who only met HDAP criterion without O-HCAP components for HCAP classification [7, 31]. On the contrary, the remaining studies enrolled patients who included the other criteria for HCAP, and reported relatively high incidence of MDR pathogens [30, 32]. In line with this concept, among 61 patients who only met HDAP criterion without the other criteria for HCAP in our study, the isolated rate of MDR pathogens was relatively low (7 patients, 11.4%). We also demonstrated that presence of O-HCAP components exhibited higher occurrence of MDR pathogens in patients with HDAP. Among the O-HCAP category, recent hospitalization and NHAP were more frequently observed in the MDR pathogens group. Especially, recent hospitalization was independently associated with the isolation of MDR pathogens in multivariable analysis. Therefore, our study found that MDR pathogen infection in HDAP could be associated with recent hospitalization, rather than HD status itself, similar to previous studies [19, 26]. The present study revealed that PSI score than 147 was also significantly associated with HDAP caused by MDR pathogens. Furthermore, the proposed prediction tool using these two risk factors showed a moderate discriminatory power for risk stratification for an infection with MDR pathogens in HDAP patients. Therefore, our findings could be helpful in physicians’ decisions to select HDAP patients who need to be treated for MDR pathogens.

The present study has several limitations. Firstly, the main limitation of this study is retrospective design. Because the effect of missing data in the results is unknown, our study may be vulnerable to selection bias. And a small sample size did not allow us to draw a robust conclusion. We were unable to enroll a large number of HDAP patients despite the fact that they were collected from three centers over 5 years. Larger studies are needed to validate our results and to strengthen the power to identify risk factors of MDR pathogens. Secondly, the microbiological etiology could be identified in only about 50% of enrolled patients. Possible reasons for low detections would include an inability to collect lower respiratory tract specimens, prior antibiotic use before specimen collection, insensitive diagnostic tests for known pathogens, a lack of testing for other recognized pathogens such as *coxiella*, unknown pathogens, and possible noninfectious causes such as aspiration pneumonitis [33]. Thirdly, median age of the patients is 71 years in the present study. Aging is associated with declines in adaptive and innate immunity [34]. Infections occur more frequently in the elderly, and the age-related remodeling of the immune system plays any role in the development of HDAP or HDAP with MDR pathogens [34]. Although age was not associated with the isolation of MDR pathogens among HDAP patients in our study, the results might be biased towards the old age population. Finally, the analysis for distribution of MDR pathogens included entire study populations with culture-negative pneumonia. Thus, we may have skewed our findings in that the true incidence of MDR pathogens could have been underestimated. In this study, we founded two independent risk factors for occurrence of MDR pathogens in patients with HDAP. And the prevalence of MDR pathogens was <10% in patients without one of these risk factors. If validated in subsequent multicenter studies, this prediction rule could potentially assist clinicians who are deciding on whether to administer anti-MRSA or anti-pseudomonal therapy to patients with HDAP.

## Conclusion

This multicenter, retrospective, observational study offers the findings of the clinical epidemiology, microbiology, and predictive factors of MDR pathogens in patients with HDAP. The HDAP concept itself as an HCAP has limited value in selecting patients harboring MDR pathogens. It could be necessary to stratify the patients with regard to risk factors in order to properly identify infection with MDR pathogens. Although large-scale prospective studies are needed to confirm our results, our findings would be helpful for physicians’ decisions to select HDAP patients harboring MDR pathogens.

## Abbreviations

*A. baumannii*: *Acinetobacter baumannii*
ATS/IDSA: American Thoracic Society/Infectious Diseases Society of America
CAP: community-acquired pneumonia
CRP: C-reactive protein
CURB-65: Confusion, Urea, Respiratory rate, Blood pressure, Age ≥ 65
ESBL: Extended-spectrum b-lactamase
HAP: Hospital-acquired pneumonia
HCAP: Healthcare-associated pneumonia
HD: Hemodialysis
HDAP: Hemodialysis-associated pneumonia
ICU: Intensive care unit
*K. pneumoniae*: *Klebsiella pneumoniae*
MDR: Multidrug-resistant
MRSA: Methicillin-resistant *Staphylococcus aureus*
NHAP: Nursing home-acquired pneumonia
O-HCAP: The HCAP other than HDAP
*P. aeruginosa*: *Pseudomonas aeruginosa*
PSI: Pneumonia Severity Index
*S. aureus*: *Staphylococcus aureus*
*S. pneumoniae*: *Streptococcus pneumoniae*
USRDS: The United States Renal Dada System
WBC: White blood cell
